# Beef Tea

**Published:** 1893-02

**Authors:** 


### BEEF TEA.

Prepared by a trained nurse is as follows:—Cut up 2 lbs. of lean, juicy sirloin steak into pieces about two inches square. Grease a saucepan lightly with butter, which place over a very hot fire of red coals, and as soon as the pan is hot toss the beef in. Turn the pieces over and over with a fork, letting them brown slightly on each side; there will be scarcely a drop of juice in the pan while this is being done, so quickly does the heat accomplish its work. As soon as the pieces are heated through, take them out one by one and rapidly squeeze them through a wooden lemon squeezer (which must be standing in boiling water) into a bowl which has been well heated. Put a small pinch of salt into the juice and cover the bowl well over to preserve the heat. This manner of preparing beef tea is most valuable when it is required at once—taking only a few minutes to make, and the entire strength of the meat being extracted.

